# Rationale and design of the GLADE study: a randomized, multicenter, double-blind, placebo-controlled trial evaluating the safety and efficacy of gestrinone subdermal bioabsorbable pellet in endometriosis-related pelvic pain

**DOI:** 10.1080/07853890.2025.2527352

**Published:** 2025-07-11

**Authors:** André Malavasi, Camilla Moreira Ribeiro, Leandro Barile Agati, Agnaldo Silva Filho, Charles Berger, Eduardo Schor, Ana Comin, Eduardo José Bezerra Neto, Taís Helena de Oliveira, Polyana Raposo Caldas, Francisco Lopes, Nathalia Westphalen, Francisleny Vieira, Eduardo Augusto Rabelo Socca, Daniele Komar, Eduardo Ramacciotti

**Affiliations:** ^a^ Department of Obstetrics and Gynecology, School of Medicine at, São Paulo State University (HC-FMUSP), São Paulo, SP, Brazil; ^b^ Science Valley Research Institute, Santo André-São Paulo, Brazil; ^c^ Department of Obstetrics and Gynecology, Federal University of Minas Gerais (UFMG), Belo Horizonte, Minas Gerais, Brazil; ^d^ Clínica Berger, Blumenau, Santa Catarina, Brazil; ^e^ Department of Gynecology, Escola Paulista de Medicina, Federal University of São Paulo (EPM-UNIFESP), São Paulo, SP, Brazil; ^f^ Clínica Saint Beauté Clinique, Balneário Camboriú, Santa Catarina, Brazil; ^g^ Instituto Ginoped São Paulo, São Paulo, Brazil; ^h^ Hapvida NotreDame Intermédica, São Paulo, Brazil; ^i^ Consultório Lopes e Sartorelli, Paraná, Brazil; ^j^ Department of Pathology, Loyola University Medical Center, Maywood, IL, USA

**Keywords:** Endometriosis, pelvic pain, subcutaneous pellet, gestrinone, R-2323

## Abstract

**Background:**

Pelvic pain secondary to endometriosis is a disabling condition. There are multiple treatments available, with variable endpoints. No prospective controlled studies were carried out evaluating subdermal pellets of gestrinone in this population.

**Methods:**

One hundred participants with documented deep infiltrative endometriosis who underwent surgery without satisfactory response will be randomly assigned (1:1) to either gestrinone 85 mg subdermal bioabsorbable pellets or placebo. Both arms will receive levonorgestrel-releasing intrauterine system (LNG-IUS 12). The treatment duration will be 6 months, with baseline, 3 months and 6 months clinical visits. The primary endpoint is a combination of serious adverse events (SAEs) accumulated within 6 months of insertion of the gestrinone or placebo pellet and collected through spontaneous reports and clinical findings. They include death, threat or risk to life, need for hospitalization, prolongation of pre-existing hospitalization, permanent disability or damage, congenital anomaly; or significant medical occurrences such as venous thromboembolism. The primary safety outcome will be the percentage of patients who do not experience SAEs 6 months after randomization. Androgenization, changes in laboratory exams and in pelvic pain intensity as well as quality of life (SF-36 and EHP-30 questionnaires) will be further evaluated. Daily data on uterine bleeding patterns and the use of pain relief medication will be remotely collected using an App. Pharmacokinetics profile of gestrinone pellet will be characterized.

**Conclusion:**

This is the first multicenter randomized controlled trial to evaluate the safety, tolerability and pharmacokinetics profile of subdermal gestrinone pellets and might inform clinical practice for treating these patients.

**Administrative Information:**

GLADE trial is registered at ClinicalTrials.gov (NCT05570786). This is an investigator-initiated research supported by Biòs Farmacêutica.

## Introduction

Pelvic pain is a disabling condition for women of reproductive age, negatively impacting their quality of life and work efficiency. Doctors frequently neglect it despite its estimated worldwide prevalence ranging from 5.6 to 26.6 [1,2]. Pelvic pain is considered a symptom of multifactorial origin in which endometriosis is the leading gynecological cause that affects 5–10% of women in their reproductive years. It means around 176 million women with endometriosis worldwide [3].

Surgical and hormonal treatments with variable endpoints are available and are still considered a significant challenge for medicine. Recommendations from different medical guidelines for managing the disease include surgery or a combination of surgical and pharmacological strategies [4–6]. Hormone therapy includes progestogens, anti-progestogens, combined oral contraceptives, gonadotrophin releasing hormone (GnRH) agonists, GnRH antagonists, the levonorgestrel intrauterine system (LNG-IUS), danazol and aromatase inhibitors. The choice of treatment for endometriosis must consider the severity of the symptoms, extent, and location of the lesions, comorbidities, age, intention to become pregnant, adverse drug events, and contraindications. Surgical treatment is indicated in the case of severe and disabling symptoms without improvement after the pharmacological treatment. However, post-surgery recurrence rate of painful symptoms is 21.5% after 2 year and 40–50% after 5 years of the surgery [4–6]. The direct costs for treatment of endometriosis are significant. The Brazilian Unified Health System (SUS) in 2021 registered 26,400 medical appointments and 8,000 hospitalizations for endometriosis with limited access to multifunctional teams specialized in endometriosis and longtime waiting for a surgery [7]. In this context, pharmacological treatment for endometriosis is prominent.

A Cochrane Review about progestagens and anti-progestagens for pain associated with endometriosis showed gestrinone as anti-progestogen (i.e. a substance that prevents cells from making or using progesterone) and one of the first drugs for the treatment of endometriosis and myomas [8]. Gestrinone acts centrally on the hypothalamic pituitary system by suppressing the release of luteinizing hormone (LH) and follicle-stimulating hormone (FSH). Both continuous progestogens and continuous gestrinone are effective therapies for the treatment of painful symptoms associated with endometriosis. There was no overall evidence of a benefit of one oral progestogen over another. However, this conclusion must be treated with caution due to the lack of placebo-controlled studies [8]. The World Endometriosis Society (WES) [5] and the European Society of Human Reproduction and Embryology (ESHRE) [6] guidelines discuss the use of gestrinone as a possible medication for the treatment of endometriosis-related pain. Gestrinone should be used for those whom other treatments have proven ineffective despite potential treatment-associated adverse effects [5]. However, there is still a lack of quality evidence from placebo-controlled randomized clinical trials.

GLADE (Gestrinone peLlet AnD Endometriosis) Study is a decentralized, patient-centered, multicenter, randomized, double-blind, parallel, placebo-controlled clinical study to primarily evaluate the safety and tolerability of subdermal gestrinone bioabsorbable pellet in the treatment of pelvic pain secondary to endometriosis. With this study, we hope to advance the knowledge about gestrinone subdermal pellets, their possible side effects, pharmacokinetics profile and their ability to alleviate pain and improve quality of life of women with endometriosis.

### Gestrinone compound profile

#### Physicochemical properties and pharmaceutical formulation

3-ethyl-17-hydroxy-18,19-dinor-17α-pregna-4,9,11-trien-20-yn-one, known as gestrinone, ethylnorgestrienone or R-2323 is a synthetic derivative of 19-nortestosterone developed in 1960 originally as a contraceptive and has been studied as a treatment for leiomyoma and endometriosis [9]. It is a compound with the molecular formula C21H24O2 and a molecular weight 308.4.14.

#### Mechanism of action

The mechanism of action of gestrinone is complex and multimediated. It inhibits folliculogenesis and acts on the hypothalamic-pituitary axis, preventing the increase in gonadotropins (FSH and LH) in the middle of the ovulatory cycle, causing estrogen inhibition, anovulation, and amenorrhea. It has an affinity for progesterone and estrogen receptors and a low affinity for androgen receptors while at the same time inducing a reduction in the expression of progesterone receptors [10]. It can reduce the production of SHBG (Sex-hormone binding globulin), which causes an increase in blood levels of free testosterone and progesterone, leading to the emergence of their androgenic effects [11].

#### Metabolization and excretion

After oral administration, gestrinone has a serum half-life of around 24 h, undergoing active hepatic metabolism essentially by hydroxylation, and its metabolic conjugates are eliminated through urine (40–45%) and feces (30–35%) [12].

#### Side effects

Most of the adverse effects of gestrinone are due to its androgenic activity and include acne, skin and hair seborrhea, mild hirsutism, edema, weight gain, voice changes, androgenetic alopecia, and clitoral hypertrophy. The drug showed an embryotoxic effect on animals, with a risk of androgenization of female fetuses. It reduces HDL concentrations, and it is recommended to monitor cholesterol and liver transaminase levels in hyperlipidemic patients and glucose levels in diabetic patients. Other effects related to inhibition of the pituitary-ovarian axis may include menstrual disorders and amenorrhea, hot flashes, sweating, breast reduction, changes in libido, vaginal dryness, and emotional fluctuations [13].

#### Clinical evidence

Previous clinical studies evaluating gestrinone and endometriosis are scarce. A study by Diaz et al. (1977) assessed the contraceptive effect of the subdermal gestrinone implant in Chilean women for 12 months, as well as the emergence of possible adverse events. The authors reported a potent inhibitory effect on fertility in patients, which was accompanied by an increase in transaminases and the appearance of acne, headaches, and hirsutism [14].

Oral treatment with gestrinone (2.5 to 5 mg twice a week) induced improvement in symptoms associated with endometriosis without the need for daily administration of the drug, which is usually required in similar conservative therapies, representing an option for treatment of the disease. Furthermore, patients treated vaginally tend to have fewer androgenic adverse events such as acne, seborrhea, and weight gain than those treated orally [15].

Worthington et al. (1993) evaluated the effects of oral gestrinone (1.25 and 2.5 mg twice a week for 6 months) on some metabolic parameters of women with laparoscopically confirmed endometriosis. The study concluded that the drug was effective in treating the disease without inducing any loss of bone density but caused unfavorable effects on serum lipids and lipoproteins that were reversed 6 months after the end of treatment [16].

A randomized clinical study involving 43 women with endometriosis compared 6 months of treatment with gestrinone (5 mg per week) or buserelin nasal spray (300 µg every 8 h) with a 5-year follow-up and found that both treatments were effective in remitting endometriosis. There were reports of acne and vascular events such as edema and heaviness in the lower limbs in the group that received gestrinone [17].

In 1996, The Gestrinone Italian Group Study compared oral treatment with gestrinone and leuprolide acetate for pelvic pain in women with endometriosis in a randomized, double-blind clinical study, concluding that both were equally effective. However, gestrinone did not affect the patient’s bone density despite inducing fluctuations in serum lipid values [18].

Trials evaluating gestrinone in the form of a gel for vaginal application (Gestrinone Pentravan^®^) for the treatment of endometriosis have also been carried out and report positive effects of the drug in relieving refractory pain [19].

Subdermal gestrinone bioabsorbable pellet could be a safe and effective alternative for treating endometriosis-associated pelvic pain and minimize adverse events observed with oral administration.

### Objectives

#### Primary objective

To evaluate the safety and tolerability of the use of gestrinone subdermal bioabsorbable pellet in women with pelvic pain secondary to endometriosis who underwent surgery without satisfactory response.

#### Secondary safety objectives

To evaluate the potential for androgenization of the use of gestrinone subdermal bioabsorbable pellet in women with pelvic pain secondary to endometriosis who underwent surgery without satisfactory response.

#### Other exploratory objectives

To compare the use of gestrinone subdermal bioabsorbable pellet versus placebo pellet on participant satisfaction, change in pelvic pain intensity, use of pain relief medication, uterine bleeding pattern and quality of life. Additionally, the pharmacokinetic profile (PK) of gestrinone will be characterized in a subgroup of participants.

## Patients and methods

The report of this study protocol follow the SPIRIT (standard protocol items: Recommendations for interventional trials) 2013 guidelines [20].

### Study design

The first participant was included on February 25th 2023 and the last patient last visit is estimated for April 2025. The laboratory analysis for measurement of gestrinone plasma levels are undergoing. We will carry a decentralized, patient-centered, multicenter, randomized, double-blind, parallel, placebo-controlled study. The study will be conducted in 7 research centers in Brazil (hospital and clinics).

**Figure 1.**
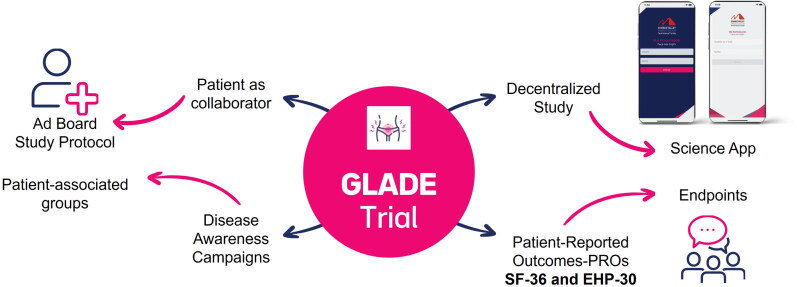
Protocol innovations.

The study protocol has several innovations to increase participant recruitment and retention such as the organization of disease awareness campaigns and the participation of a patient representative as member of the Trial Steering Committee. Participant representative provided information about the burden of the disease and the impact of the treatment through a patient perspective that enabled the inclusion of patient-reported outcomes in the study protocol. An App will be used to remotely collect diary registries about uterine bleeding pattern and use of analgesics for pain relief and other adverse events in real time. These protocol innovations are depicted in Figure 1.

The study protocol was approved by the Institutional Review Board (IRB – Comitê de Ética Hospital Leforte, CAAE: 64457622.5.1001.5485, Approval Number: 5.748.523, Dated: November 9^th^, 2022). The study protocol was approved by the institutional review boards at all research sites. Written informed consent will be obtained from all patients before any study procedure is applied. The study was registered at ClinicalTrials.gov (NCT05570786). The first participant was included on February 25^th^ 2023 and the last patient last visit is estimated for April 2025.

**Figure 2.**
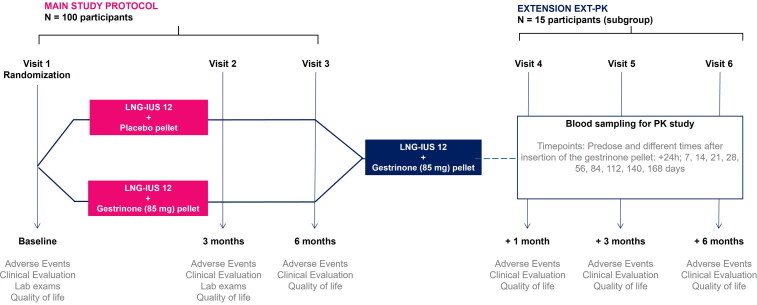
Study design.

The study protocol is composed of two stages: 1) Main protocol: randomized, double-blind, parallel, placebo-controlled, *N* = 100 participants, treatment duration and follow-up: 6 months; 2) Extension-PK (EXT-PK): open-label, *N* = 15 participants (subgroup of participants who concluded the main protocol and accepted to receive the treatment; follow-up: 6 months. The study design is depicted in Figure 2.

Participants eligible for the main stage of the study will be randomized into one of the treatment arms:

- Experimental: Insertion of the levonorgestrel-releasing intrauterine system (LNG-IUS 12, Kyleena^®^) and gestrinone subdermal bioabsorbable pellet (85 mg)
- Comparator: Insertion of the levonorgestrel-releasing intrauterine system (LNG-IUS 12, Kyleena^®^) Kyleena^®^ levonorgestrel-releasing intrauterine system and subdermal implant of placebo bioabsorbable pellet

The insertion of the levonorgestrel-releasing intrauterine system (LNG-IUS 12, Kyleena^®^) is necessary as a contraceptive method for all study participants. LNG-IUS (LNG-IUS 12) has been developed for up to 5 years of use with an average release rate of ∼12 μg/24 h over the first year [21]. Clinical assessments will be recorded at recruitment (baseline), 3 and 6 months after pellet insertion. Assessment of the primary outcome will be carried out 6 months after insertion of the gestrinone pellet.

Participants will be invited to an extension phase (open-label) of the study to provide additional long-term safety data (EXT-PK). A subgroup of 15 participants at the end of 6 months of the main stage of the study who accept to participate in the extension phase will receive an insertion of a gestrinone pellet (85 mg), and the serum concentration of gestrinone will be monitored to characterize its pharmacokinetic profile (PK). Clinical assessments will be recorded at recruitment (baseline), 1, 3 and 6 months after pellet insertion. Blood sampling for PK study will be conducted.

Bioabsorbable gestrinone pellets are produced and marketed by Biòs Farmacêutica following standardized procedures and current legislation. Pellets containing a placebo will be prepared under the same conditions as those containing the product ingredient. Biòs Farmacêutica will donate all pellets used in this study.

GLADE is an investigator-initiated project. Biòs Farmacêutica funded the trial but was not involved in the study design, data collection, analysis, interpretation, writing of the manuscript, or decision to publish. The Trial Steering Committee designed the study protocol and is monitoring the trial’s progress. The first draft of the manuscript was written by the first and the last author and revised based on comments from all the authors. No writing assistance was provided.

### Patients

The study will enroll 100 women with refractory pain control who underwent surgery for endometriosis at least 3 months ago and who had a histologically confirmed diagnosis of deep infiltrative endometriosis. Eligible participants are women with pelvic pain secondary to endometriosis surgically treated with refractory symptoms, independent of pain intensity. .Endometriosis-associated pelvic pain includes dysmenorrhea, dyspareunia, dysuria, dyschezia, and nonmenstrual pelvic pain.

Study participants have a medical history of indication for surgery as an option to reduce endometriosis associated-pain after having been failed to standard hormone treatment (combined hormonal contraceptives, progestogens, GnRH agonists or GnRH antagonists). Surgeries were conducted to fully eliminate endometriotic lesions from both the posterior compartment and anterior compartment, as well as from bowel compartment whenever present.

Detailed eligibility criteria are listed in Table 1.

**Table 1.** Study eligibility criteria.

Inclusion criteria
1. Willingness to provide informed consent
2. Woman aged between 18 and 50 years
3. Body weight between 50 ± 5 kg and 90 ± 5 kg
4. Pelvic pain secondary to endometriosis surgically treated with refractory symptoms, independent of pain intensity
5. Deep infiltrative endometriosis documented by biopsies (histopathological examination)
6. Last endometriosis surgery at least 3 months before randomization
7. Not planning to become pregnant within 12 months after the screening visit or be surgically sterilized
8. Absence of changes in the breast (BI-RADS 1 and BIRADS-2 classification) documented by an imaging report (mammogram for women aged > 40 years or bilateral breast ultrasound for women aged < 40 years) performed less than 12 months before randomization
9. Agreement not to use other hormones (estrogens, androgens and progestins) in any pharmaceutical form during the study
Exclusion criteria
1. Chronic severe disorders, including metastatic malignancies, end-stage renal disease with or without dialysis, clinically unstable heart disease, or any other disorder that, in the opinion of the investigator, excludes the participant from the study
2. Suspected or confirmed diagnosis of immunodeficiency based on medical history and/or physical or laboratory examination
3. Other medical or psychiatric conditions, including recent laboratory abnormalities (within the last 12 months) that may increase risks to the study participant or, at the discretion of the investigator, make the participant inappropriate for the study
4. Personal history of thromboembolic events
5. Use anticoagulant medication
6. Contraindication to the use of hormonal contraceptives
7. Suspected or confirmed pregnancy
8. Breastfeeding
9. Current or recurrent pelvic inflammatory disease or other conditions that increase the risk of pelvic infections
10, Postpartum endometritis or septic miscarriage in the last 3 months
11. Abnormal uterine bleeding of unknown etiology
12. Congenital or acquired uterine anomalies, including fibroids (leiomyomas or fibromas) that cause distortion of the uterine cavity
13. Uterine or cervical malignancy
14. Suspected or confirmed diagnosis of estrogen-dependent neoplasm, including breast cancer
15. Cervicitis or vaginitis, including bacterial vaginosis or another uncontrolled lower urinary tract infection
16. Cervical dysplasia
17. Active liver disease or dysfunction
18. Benign or malignant liver tumors
19. Allergy or intolerance to levonorgestrel, gestrinone or any other ingredient or component of the Kyleena^®^ formulation or hormonal pellets
20. Previously inserted intrauterine device or levonorgestrel-releasing intrauterine system that has not been removed
21. History of recent trophoblastic disease and continued high HCG levels
22. Bacterial endocarditis
23. Hyperandrogenism at the time of randomization, defined by: hirsutism: Ferriman-Gallwey score ≥ 8; clitoromegaly: defined by the Clitoral index ≥ 35 mm^2^, acne: defined by the IGA scale (Investigator’s global assessment) grade 5 – severe inflammatory acne dominates the area and there is a large number of comedones, pustules, papules and cystic acne; alopecia with sequelae of scalp thinning
24. Diagnosis of polycystic ovary syndrome
25. Participation in another pharmacotherapeutic or investigational medical device study within 30 days prior to the start of study treatment
26. Tobacco Use
27. Use of testosterone-derived hormones and analogues in the last month

Potential participants will be recruited by publicizing the study by doctors in their clinics, through social media, and the Science Valley Research Institute website in the study tab by answering a volunteer questionnaire. All the materials for recruitment purposes will be approved by the local IRB.

### Discontinuation criteria

Statement by the participant or their legal representative requesting discontinuation of participation in this study. Or adverse events, symptoms, and signs of toxicity at the investigator’s discretion. Or any condition in which, in the opinion of the investigator, it would be advantageous for the participant not to comply with the procedures specified in this protocol and by the sponsor’s request. In case of premature discontinuation, a clinical visit will be conducted, and the same date of the last visit (6-month) will be collected.

An Independent Data Safety Monitoring Board of academic physicians not associated with the study sponsor will provide data and safety adjudgment. A Clinical Events Classification Committee will give a blinded adjudication of clinical events.

## Study endpoints

### Primary safety outcome

It is a combination of serious adverse events (SAEs) accumulated within 6 months of insertion of the gestrinone or placebo pellet and collected through spontaneous reports and clinical findings. The primary safety outcome will be the percentage of patients who do NOT experience SAEs 6 months after randomization.

SAEs are unfavorable medical events that result in any of the following outcomes:

1. Death
2. Threat or risk to life
3. Need for hospitalization
4. Prolongation of pre-existing hospitalization
5. Permanent disability or damage
6. Congenital anomaly; or
7. Significant medical occurrences that, based on appropriate medical judgment, may harm the participant and require medical or surgical intervention to prevent any of the other occurrences mentioned. In this study, the occurrence of thromboembolic events (such as, for example, acute myocardial infarction, pulmonary embolism, deep vein thrombosis, ischemic stroke, and transient ischemic attack) was pre-defined as a significant medical occurrence.

### Secondary safety outcomes [timepoints: pre-pellet insertion assessment, 3 and 6 months after gestrinone or placebo pellet insertion]

The main secondary safety outcome will be defined as the number of participants who experience androgenization defined by:

1. Hirsutism: defined by Ferriman-Gallwey Score ≥ 8
2. Clitoromegaly: defined by Clitoral index ≥ 35 mm^2^
3. Acne: defined by the IGA scale (Investigator’s global assessment) grade 5 – severe inflammatory acne dominates the area, and there are many comedones, pustules, papules, and cystic acne
4. Alopecia with sequelae of scalp thinning
5. Deepening of the voice

### Other secondary safety endpoints [timepoints: pre-pellet insertion assessment and 3 months after gestrinone or placebo pellet insertion]

The number of participants who experience:

1. Laboratory values outside the reference range and clinically relevant for serum hormone concentrations (total testosterone, free testosterone, and SHBG)
2. Clinically significant change in total cholesterol, HDL-C, VLDL-C, and triglycerides from baseline
3. Hematological disorders with recent onset, defined as: decreased lymphocyte count < 500/mm3 (or < 0.5 × 109/l); decreased neutrophil count < 500/mm3 (or < 0.5 × 109/l); decreased platelet count < 30,000/mm3 (or < 30.0 × 109/l); and anemia with decreased Hb < 7.0 g/dl (or < 4.35 mmol/l)
4. Hepatic adverse events (increase in ALT or AST > 3 times the ULN or baseline values or total bilirubin > 2 times the ULN or baseline values or suspected hepatocellular or cholestatic hepatotoxicity)
5. Renal adverse events e.g. confirmed increase in serum creatinine ≥ 1.5 times the ULN or baseline values; a clinically significant decrease in creatinine clearance; a clinically significant increase in serum urea; and new onset of proteinuria

### Exploratory efficacy endpoints [timepoints: pre-pellet insertion assessment, 3 and/or 6 months after gestrinone or placebo pellet insertion]

Exploratory efficacy endpoints are defined as:

1. Median participant satisfaction (ranging from 1 to 5, from very satisfied to very dissatisfied) – assessment
2. Median intensity of pelvic pain and dysmenorrhea assessed by the VAS scale
3. Proportion of participants who used medication to relieve pelvic pain
4. Median number of days that participant used medication to relieve pelvic pain
5. Change in uterine bleeding pattern
6. Proportion of participants with a change in Health-related Quality of Life measured with the 36-item Medical Outcomes Study Health Survey Form (SF-36)
7. Average change in total score and score for each domain of the 36-item Medical Outcomes Study Health Survey Form (SF-36)
8. Proportion of participants with changes in the Endometriosis Health Profile 30 (EHP- 30 The Endometriosis Health Profile) score
9. Average change in the total score and the score of each domain of the Endometriosis Health Profile 30 (EHP- 30 The Endometriosis Health Profile)

### Other exploratory outcomes

The pharmacokinetic profile of gestrinone will be characterized in a subgroup of participants (*N* = 15). The pharmacokinetic parameters will be determined: area under the curve (AUC(0-∞)), maximum concentration (Cmax), time until reaching maximum concentration (Tmax), and half-life (t1/2).

### Assessment of outcomes measures

Outcomes measures will be assessed during each of the three study visits in the main study protocol (randomized, double-blind, parallel, placebo-controlled stage), including a baseline visit (pre-treatment), a 3-month visit and 6-month visit. A trained team, including a gynecologist, will conduct the study in the outpatient setting in all study centers. Clinical assessments, monitoring of adverse events, evaluation of androgenization (hirsutism, clitoral index, acne, alopecia, and deepening of the voice), as well as pelvic pain intensity and quality of life assessed by SF-36 questionnaire will be evaluated at all the three study visits. Laboratory blood assessment will be conducted at the baseline visit and in the 3-month visit and include: complete blood count, liver and kidney function (AST, ALT, alkaline phosphatase, urea, creatinine, and uric acid), lipid profile (triglycerides, total cholesterol, HDL, LDL, VLDL) and blood glucose, coagulogram (PT and aPTT), and plasma hormone levels (total testosterone, free testosterone, and SHBG). Quality of life assessment by the EHP-30 questionnaire will be applied at the baseline visit and in the 3-month visit. Data on uterine bleeding pattern and use of medication to relieve pelvic pain will be remotely collected using and electronic diary available at the App used in the study protocol.

Outcomes measures in the EXT-PK (open-label) will be assessed during each of the four study visits in which clinical evaluation, monitoring of adverse events, evaluation of androgenization, pelvic pain intensity and quality of life by SF-36 questionnaire will be conducted in each of the clinical visits, including baseline (pre-treatment), 1-month, 3-month, and 6-month after the insertion of gestrinone subdermal bioabsorbable pellet. Blood sampling for PK study will be conducted: pre-insertion of the gestrinone pellet (predose); different times after insertion of the gestrinone pellet: +24h; 7, 14, 21, 28, 56, 84, 112, 140, 168 days. Data on uterine bleeding pattern and use of medication to relieve pelvic pain will be remotely collected using an electronic diary established to be available at the App established to be used in the study protocol.

### Study procedures

Participant-reported outcomes will be collected *via* the App used in the study protocol: Quality of Life 36-item Form Health Survey (SF-36); endometriosis health profile – Endometriosis Health Profile 30 (EHP-30); diary to record the use of analgesics for pelvic pain and uterine bleeding pattern. On the first visit, access to the App will be provided to the participant; the participant will receive guidance on how to use the App, and baseline data will be collected. Following the study procedure schedule, participants will receive reminders through the App to record outcomes, and telephone contact will be made for those who do not complete the form regularly. At follow-up visits, participants will be retrained if they present difficulties in using it.

## Screening procedures

### Gynecological assessment

A gynecological assessment will be carried out to check the conditions of the female reproductive tract and the suitability for the insertion of the levonorgestrel-releasing intrauterine system (LNG-IUS 12). Furthermore, menstrual history, hormone use, pregnancy, and fertility history will be collected. A transvaginal ultrasound will be performed to assess the positioning of the device.

### Treatments

#### Insertion of the levonorgestrel-releasing intrauterine system (LNG-IUS 12)

The LNG-IUS 12 (Kyleena^®^) must be inserted in an outpatient setting by the investigator following the manufacturer instructions. After carrying out a gynecological examination to check the size and position of the uterus, the cervix should be visualized with the aid of a speculum, and the cervix and the vagina should be cleaned with an antiseptic solution. With the help of the sliding guide, the IUS inserter must be pushed to the bottom of the uterus to position the device.

#### Insertion of the investigational product or subdermal bioabsorbable placebo pellet

The investigator will conduct the procedure in an outpatient setting. After local anesthesia, a small incision (4–5 mm) will be made, through which the pellet will be inserted under the skin of the buttock using a trocar. It will be performed just after the insertion of the LNG-IUS 12, and the randomization procedure.

### Safety and efficacy endpoint assessments

Suspected events and outcomes must be analyzed by the Clinical Events Classification Committee. Safety results should be indicated on the eCRF clinical status page and should be submitted for review.

### Laboratory evaluation

Blood samples (total volume of 10 mL) will be collected through venipuncture from the participant in the following tubes: tubes containing EDTA anticoagulant to perform the blood count, tubes with separating gel (to evaluate biochemical parameters of liver and kidney function, hormonal and lipid profile) or citrate (to perform a coagulogram), observing a prior 8-hour fast. After separating the serum, dosages will be carried out using commercially available kits following the instructions specified by the manufacturer. The local laboratory will conduct the analyses using its protocols and equipment and following Biosafety Standards and Good Laboratory Practices.

## Assessment of androgenization

### Hirsutism

The investigator will assess the degree of hirsutism. The participant should be instructed to suspend hair removal/waxing for approximately 14 days before each visit. The use of lasers for hair removal will not be permitted during the entire study period. To evaluate and quantify hirsutism, the modified Ferriman-Gallwey Scoring System will be used, which includes 9 body regions (excluding the legs and armpits) for access to hair growth, grading from 0 (no terminal hair growth) to 4 (extensive hair growth) in 9 locations. The total score ranges from 0 to 36, with a score greater than 8 considered a sign of androgen excess in Caucasian women. A score of 8 – 15 indicates mild hirsutism; above 15, moderate to severe hirsutism. For other ethnic groups, the amount of hair expected for each specific ethnicity must be considered [22].

### Clitoris measurement

The investigator will measure the participant’s clitoris (portion corresponding to the glans) [23]. The size of the clitoris will be measured using a 15 cm plastic ruler. The Clitoridean Index will be considered as the product of the longitudinal diameter (in millimeters) x transverse diameter (in millimeters), considering clitoromegaly when these measurements are more significant than 6.5 and 4.4 respectively, and the Clitoridean Index greater than 35 mm^2^ [24]. Additionally, the participant’s clitoris corresponding to the part of glans + corpus will be measured and registered.

### Deepening of the voice

A speech therapist will evaluate the participant’s vocal pattern using protocols and equipment validated by evaluating professionals. The participant’s voice will be recorded using a digital recorder for evaluation. The pattern observed on each visit will always be compared with that recorded during the initial visit (Basal) to detect possible vocal changes and, more specifically, the deepening of the voice.

### Assessment of the degree of acne

The severity of acne will be assessed

By using the IGA scale (Investigator’s global assessment) to be used by the trained researcher and described below:

0 – Clear skin: no evidence of acne
1 – Almost clear skin: some inflammatory lesions and some non-inflamed papules present (developing papules, without redness yet)
2 – Moderately severe acne: some non-inflammatory lesions dominate the area with some pustules and papules (there are no cystic lesions)
3 – Non-inflammatory acne dominates the area, and some inflammatory lesions can be found. There may or may not be a small cystic lesion in the area,
4 – Inflammatory and non-inflammatory acne lesions are more visible. There may or may not be some cystic acne lesions.
5 – Severe inflammatory acne dominates the area. Many comedones, pustules, papules, and cystic acne will be present.

## Alopecia

The investigator will assess the occurrence of alopecia according to the presence of rarefaction sequelae on the scalp.

### Uterine bleeding or bleeding-free days

The participant must record in the electronic diary (App) whether there has been uterine bleeding in the last 24 h. If the answer is positive, she must inform us of the intensity of the bleeding (whether spotting or bleeding). Based on the data documented in the participant’s diary, the following parameters will be evaluated:

Total number of days of amenorrhea
Total number of bleeding-free days in the reference period
Total number of days of bleeding and spotting (escape)
Total number of days with bleeding or spotting in the reference period
Number of consecutive bleeding-free days
Number of consecutive days without bleeding after starting treatment
Total number of days with spotting (escape)
Total number of days with spotting (escape) in the reference period
Total number of days to cessation of bleeding after starting treatment

The bleeding pattern will be classified according to the criteria of Belsey [25] described below: normal bleeding − 3 to 5 episodes of spotting or bleeding within 90 days; prolonged bleeding – more than 14 days of continuous spotting or bleeding within 90 days; frequent bleeding – more than 14 days of constant spotting or bleeding within 90 days; infrequent bleeding – less than 3 episodes of spotting or bleeding within 90 days; amenorrhea – no bleeding during the 90 days of the reference period.

### Assessment of changes in the intensity of pelvic pain and dysmenorrhea based on the Visual Analogue scale (VAS/EVA)

The study participants will receive a diagram based on the Visual Analogue Scale shown below to assess chronic non-cyclical pelvic pain and dysmenorrhea. The VAS scale is standardized from 0 (no pain) to 10 (in centimeters) (the worst pain imaginable).

### Record of pre-study and concomitant medications

At the screening visit, pre-study medication use will be recorded. The participant must record in the electronic diary through the App used in the study about the use of concomitant medications, especially for pain relief (analgesics).

### Assessment of patient-reported quality of life

The Medical Outcomes Study 36-item Form Health Survey (SF-36) measured health-related quality of life. Participants must respond to the SF-36 questionnaire. The SF-36 is a multidimensional questionnaire containing 36 items distributed into 8 components: functional capacity, physical aspects, pain, general health status, vitality, social and emotional aspects, and mental health. It has a final score that ranges from 0 to 100, where 0 corresponds to the worst general health status and 100 to the best health status [26].

## Endometriosis health profile 30 (EHP-30)

Participants must respond to the EHP-30 questionnaire. The EHP-30 is a self-report instrument that measures how endometriosis affects a woman’s health status and quality of life. It consists of a central questionnaire composed of 30 items that access 5 dimensions: pain, control and helplessness, emotional well-being, social support, and self-image; and a modular questionnaire with 23 items distributed across 6 scales: sexual relations, work, medical profession, infertility, relationship with children and treatment. Each scale is transformed into a score from 0 to 100, with the lowest score meaning a better quality of life [27].

### Patient satisfaction with treatment

Participants must rate their satisfaction with the treatment by choosing one of the following alternatives presented in written form (ranging from 1 to 5): very satisfied, satisfied, not sure, dissatisfied, or very dissatisfied.

### Pharmacokinetic profile of gestrinone

Blood sampling will be conducted at pre-insertion of the gestrinone pellet; different times after insertion of the gestrinone pellet: +24h; 7, 14, 21, 28, 56, 84, 112, 140, 168 days

A sensitive and specific high performance liquid chromatography-electrospray tandem mass spectrometric (LC-MS/MS) method was developed and validated by a local specialized laboratory for the determination of gestrinone in human serum using nomegestrol acetate-d5 as an internal standard. The limit of quantification (LOQ) was 0.05 ng/mL.

The pharmacokinetic parameters will be evaluated: area under the curve (AUC(0-∞)), maximum concentration (Cmax), time until reaching maximum concentration (Tmax), and half-life (t1/2).

### Reports of pregnancy

Participants should be instructed to notify the investigator immediately if they become pregnant during the study. The investigator must report any pregnancy in study participants that occurs within 30 days of the last administration of the investigational product.

### Randomization and allocation concealment

Participants will be randomized into one of the study arms in a 1:1 ratio to receive treatment with gestrinone or placebo, stratified by center. According to an electronically predefined randomization list, participants will be assigned to a treatment group (RedCap, version 11.0.3). Participants are randomly allocated to one of the study arms, and neither the investigator nor the study participants influence the allocation of participants to the groups (blinding).

### Statistical analysis

Statistical analysis will be carried out by researchers independently. Summaries by treatment group will be provided using appropriate descriptive statistics for all study variables. Descriptive statistics such as mean ± standard deviation median (minimum-maximum) will summarize continuous variables. Categorical variables will be summarized as the proportions of each category. Graphical data presentation can also be used to summarize data. The values of the scores and indices will be compared with the baseline values (baseline = pre-treatment) using the paired t-test and Wilcoxon test. Changes in laboratory measurements and other secondary outcomes will be compared with the paired Wilcoxon test. Unless otherwise indicated, all statistical tests will be interpreted at a nominal (i.e. no multiplicity adjustment) two-tailed significance level of 0.05 and all CIs at a nominal two-tailed 95% level. The safety and exploratory efficacy outcomes results will be used for analyses.

The Intention-To-Treat (ITT) and Per Protocol (PP) population will assess all safety and exploratory efficacy endpoints. The ITT population consists of all randomized participants who received the subdermal implant. The PP population includes only participants who complete all research procedures and do not present significant protocol violations, including breach of inclusion criteria.

## Discussion

Pelvic pain is of multifactorial origin, in which endometriosis is the leading cause. It is challenging to treat endometriosis and there are surgical and hormonal treatments available with variable endpoints. Post-surgery recurrence rate of painful symptoms is high and alternative hormonal methods are needed. Gestrinone is one of the clinical options to reduce endometriosis-associated pelvic pain, but data from clinical controlled trials, particularly with subcutaneous pellets, are scarce.

This will be the first multicenter randomized controlled trial to compare the safety of gestrinone subdermal bioabsorbable pellet versus placebo in women with endometriosis who underwent surgery without satisfactory response. This is the first to thoroughly evaluate the possible benefits of such a novel gestrinone pharmaceutical formulation with low-dose levonorgestrel-releasing intrauterine system (LNG-IUS 12) and might improve clinical outcomes in this population.

The study protocol has several innovations such as the participation of a patient representative as member of the Trial Steering Committee. Participant representative provided information about the burden of the disease and the impact of the treatment through a patient perspective that enabled the inclusion of patient-reported outcomes in the study protocol. The trial will use advanced technology for data collection to capture participant′s responses, as well as clinical and biological data. In addition, the study includes a variety of biochemical analyses, including an extension phase that will provide crucial information on the long-term use of gestrinone pellets and PK analyses by LC-MS/MS.

Since it is a safety-focused study, the trial has an experienced and independent Data Safety Monitoring Board and Clinical Events Classification Committee to evaluate all possible events blindly.

We acknowledge some limitations of the study. Given the possible 10% dropout rate, the sample size might be small. However, given that it is a safety-focused phase II study, results might serve as a basis for large clinical trials powered enough to conclude the safety and efficacy of gestrinone pellets in patients with pelvic pain due to endometriosis. Another limitation of the GLADE trial was not excluding concomitant adenomyosis at screening. There is a need for future studies to evaluate the efficacy of gestrinone with more precise endometriosis phenotype including concomitant adenomyosis.

In addition, the target population of GLADE trial is women with endometriosis-associated pelvic pain refractory to surgical treatment, independent of the surgical approach. The potential heterogeneity concerning surgical approaches, preferences and techniques performed at multiple Brazilian centers of expertise in endometriosis will be equilibrated between treatment groups by electronic randomization. Further larger studies addressing the efficacy of subdermal pellet of gestrinone and the potential differences of this effect on anterior, posterior or bowel compartment as well as on adenomyosis would bring evidence of a therapeutic option for pelvic pain.

## Conclusion

Gestrinone is used for the treatment of pain in women with endometriosis. Still, there is a lack of data from well-designed randomized clinical trials evaluating such a strategy using a subdermal pellet with a particular focus on safety. We expect the current study will provide high-quality evidence of therapeutic efficacy and foster future studies to further characterize subdermal pellets of gestrinone as a therapeutic option for endometriosis-related pelvic pain.

## Supplementary Material

Supplementary Materials GLADE_SPIRIT_Checklist.pdf

## Data Availability

Data sharing does not apply to this article as no new data were created or analyzed in this publication.
